# Ultrasonographic assessment of pulmonary and central venous congestion in experimental heart failure

**DOI:** 10.1152/ajpheart.00735.2023

**Published:** 2023-12-15

**Authors:** Niklas Hegemann, Pengchao Sang, Jonathan H. Kim, Ceren Koçana, Noor Momin, Jan Klages, Mariya M. Kucherenko, Christoph Knosalla, Benjamin O’Brien, Szandor Simmons, Matthias Nahrendorf, Wolfgang M. Kuebler, Jana Grune

**Affiliations:** ^1^Department of Cardiothoracic and Vascular Surgery, Deutsches Herzzentrum Der Charité (DHZC), Berlin, Germany; ^2^Charité-Universitätsmedizin Berlin, corporate member of Freie Universität Berlin and Humboldt-Universität zu Berlin, Berlin, Germany; ^3^Institute of Physiology, Charité-Universitätsmedizin Berlin, corporate member of Freie Universität Berlin and Humboldt-Universität zu Berlin, Institute of Physiology, Berlin, Germany; ^4^German Centre for Cardiovascular Research, Berlin, Germany; ^5^Center for Systems Biology, Massachusetts General Hospital and Harvard Medical School, Boston, Massachusetts, United States; ^6^Department of Cardiac Anesthesiology and Intensive Care Medicine, Deutsches Herzzentrum Der Charité, Berlin, Germany; ^7^William Harvey Research Institute, London, United Kingdom

**Keywords:** experimental heart failure, lung ultrasound, pulmonary congestion, vena cava, venous congestion

## Abstract

Pulmonary and systemic congestion as a consequence of heart failure are clinically recognized as alarm signals for clinical outcome and mortality. Although signs and symptoms of congestion are well detectable in patients, monitoring of congestion in small animals with heart failure lacks adequate noninvasive methodology yet. Here, we developed a novel ultrasonography-based scoring system to assess pulmonary and systemic congestion in experimental heart failure, by using lung ultrasound (LUS) and imaging of the inferior vena cava (Cava), termed CavaLUS. CavaLUS was established and tested in a rat model of supracoronary aortic banding and a mouse model of myocardial infarction, providing high sensitivity and specificity while correlating to numerous parameters of cardiac performance and disease severity. CavaLUS, therefore, provides a novel comprehensive tool for experimental heart failure in small animals to noninvasively assess congestion.

**NEW & NOTEWORTHY** As thorough, noninvasive assessment of congestion is not available in small animals, we developed and validated an ultrasonography-based research tool to evaluate pulmonary and central venous congestion in experimental heart failure models.

## INTRODUCTION

Advanced left heart failure (HF), caused by ischemic heart disease or valvular disease, is associated with combined forward and backward failure resulting in a downward congestive spiral, culminating in volume overload and eventually premature death (1, 2). Backward failure due to left ventricular (LV) dysfunction causes blood to back up into the left atrium, further propagating into the pulmonary circulation, as evident by increased pulmonary pressure and type 2 pulmonary hypertension (PH) (3). Inevitably, increased pulmonary vascular resistance entails increased right ventricular (RV) afterload. The RV can transiently cope with increased afterload through mechanisms like hypertrophy by using its functional reserve, but will ultimately also transition into failure (4). At the state of biventricular failure, backward congestion propagates into the venae cavae and, ultimately, the whole central venous system, marking the onset of decompensation in advanced HF.

Congestion is routinely monitored in patients with overt HF as a predictor of poor prognosis and mortality. Ultrasound is the key diagnostic tool in the diagnosis and surveillance of patients with HF. In patients, ultrasound may also be used to image structures beyond the heart to visualize congestion (5). Across the ultrasound repertoire, two tools are commonly used to assess pulmonary and venous congestion in patients with HF: *1*) lung ultrasound (LUS), a rapid, cheap, and easy-to-handle tool assessing pleural effusion, pulmonary edema, and other pathologies with partially superior diagnostic accuracy compared to chest X-ray (6, 7) and *2*) ultrasonic evaluation of the inferior vena cava (IVC), used to assess IVC dilatation, a sign of central venous congestion, usually caused by RV failure (8, 9).

Yet, the gold standard technique for the assessment of pulmonary edema in small animals is the postmortem gravimetric measurement of lung water content, preventing the possibility of serial and longitudinal study designs. Only recently have we and others started to exploit LUS’ diagnostic power as a surrogate measure for lung water content in small animals (10–12), but a robust and standardized ultrasonographic tool to assess congestion beyond the lungs in experimental HF is so far missing. Based on this, we hypothesized that the combination of LUS signs and IVC imaging would have superior performance in assessing congestion in experimental HF compared with LUS alone. To validate ultrasonography’s power in diagnosing congestion, we used two standard models of advanced HF in a retrospective analysis: rats with supracoronary aortic banding (AoB) and mice with myocardial infarction (MI). As a result, we introduce here a novel species-specific scoring system for the noninvasive assessment of congestion in mice and rats, hereinafter referred to as CavaLUS.

## MATERIALS AND METHODS

All animal experiments were approved by the local authorities (G0239/16, G0030/18, 2020N000070) and performed in agreement with the Animal Research: Reporting of In Vivo Experiments (ARRIVE) guidelines and guidelines for the Care and Use of Laboratory Animals (National Institute of Health, Bethesda, MD). Animals were housed in a 12-h:12-h light/dark rhythm with free access to food and water. To yield high power within our retrospective data analyses, we pooled data from HF models with a broad range of disease stages, including AoB rats 3, 5, or 9 wk after surgery and MI mice 6 wk after surgery with distinct genetic cardiac predisposition (C57BL/6, CCR2^−/−^, CX3CR1^−/−^, TNF-α^−/−^) with or without therapeutic intervention.

### Aortic Banding Model (Rat)

HF was induced in 4- to 8-wk-old male Sprague–Dawley rats (∼100 g body wt) by AoB surgery under ketamine-xylazine (87/13 mg/kg body wt) anesthesia and mechanical ventilation following tracheotomy (13). In brief, a metal clip was surgically implanted just before the brachiocephalic artery, leaving a remaining aortic diameter of ∼0.8 mm. Sham animals were subjected to the same surgical procedure without clip placement. Echocardiographic evaluation took place 3, 5, or 9 wk after surgery.

### Myocardial Infarction Model (Mouse)

MI was induced by permanent ligation of the left anterior descending (LAD) artery as described previously (14). In brief, 8- to 15-wk-old male (*n* = 9) and female (*n* = 25) mice were anesthetized with isoflurane and mechanically ventilated following endotracheal intubation. After left thoracotomy, the LAD was permanently ligated using a nonresorbable 8-0 suture thread. For controls, we used data from a total of *n* = 33 male and *n* = 16 female age-matched mice.

### Echocardiography

Mice and rats were initially anesthetized with 3%–4% isoflurane, which was reduced to 1.5%–2% to maintain anesthesia. Animals were placed on a heating pad to maintain body temperature and heart rates at physiological and comparable ranges. Image acquisition was performed using an ultrahigh frequency linear array transducer (MX250, 15–30 MHz or MX400, 20–46 MHz) in combination with a Vevo3100 high-resolution imaging system (FUJIFILM VisualSonics, Toronto, ON, Canada). A conventional echocardiographic evaluation was carried out in parasternal long- and short-axis views, aortic arch and four-chamber view, recording B- and M-mode, pulsed-wave, and tissue-Doppler cine loops as described previously (11, 15). Images were stored as raw data in DICOM format for offline analysis.

### IVC Imaging

Image depth was adjusted to animal size. The transducer was placed perpendicular on the midsternal line just below the xyphoid process and then slowly moved caudally until three landmarks became visible: *1*) the IVC in the lower left quadrant of the image, *2*) the portal vein located oblique above the top right border of the IVC, and *3*) the pulsatile abdominal aorta located in the lower right quadrant. B-mode images were acquired before switching to M-mode by placing the sample volume across the maximum diameter of the IVC (Supplemental Videos S1–S4). M-mode recordings were used to assess maximum IVC diameters in offline analysis.

### LUS Imaging

Sonographic evaluation of the lungs has been performed as described previously (10, 12) by placing the transducer on the right anterior axillary line, angled to 45° to point at the right lung. Once the pleura appears as white, hyperechoic horizontal line in the upper third of the image, three B-mode cine loops were recorded with a standardized gain of 20 dB and length of 3 s to ensure capturing at least one respiratory cycle (Supplemental Videos S4–S8).

### Image Analysis

Evaluation of echocardiographic, lung ultrasound, and vena cava recordings has been performed using the Vevo LAB V3.1.1 software (FUJIFILM VisualSonics, Toronto, ON, Canada). CavaLUS scoring was conducted by three independent observers (ultrasound experience, >1 year). A detailed description of individual LUS and IVC parameters and their scoring can be found in Supplemental Table S1.

### Necropsy

After euthanasia by anesthetic overdose and subsequent exsanguination or cervical dislocation, the hearts and lungs were explanted for gravimetric analysis.

### Statistical Analysis

Statistical analysis was carried out using GraphPad Prism V10.0.2 (GraphPad Software, La Jolla, CA). Data are presented as means ± SD. Individual sample sizes are given in the figure legends. Experimental data were assessed for Gaussian distribution by Shapiro–Wilk test. Normally distributed data were investigated by unpaired *t* tests, whereas not-normally distributed data were analyzed using the Mann–Whitney test. *P* values < 0.05 were considered statistically significant. For evaluation of the best-performing ultrasound scores, Pearson’s correlation, receiver-operator characteristics (ROC), and analysis of Youden’s J were conducted. Blant–Altman plots were prepared, indicating bias and 95% limits of agreement for observer comparisons.

## RESULTS

**Figure 1.**
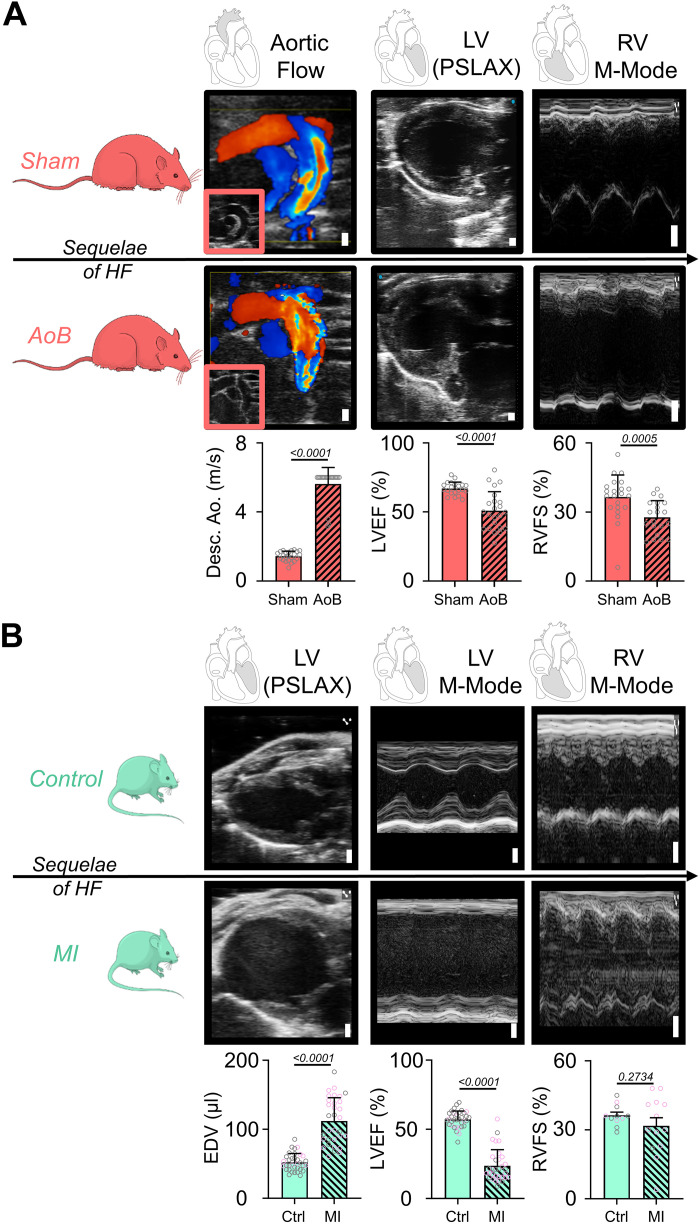
Aortic banding and myocardial infarction heart failure phenotyping. *A*: pathophysiological sequelae of heart failure in the aortic banding rat model (AoB). Color-Doppler recordings of the aortic arch with pulsed-wave Doppler examinations of the descending aorta (Desc. Ao.) flow velocities (detection limit 6 m/s), parasternal long-axis (PSLAX) B-mode, and RV M-mode images with evaluation of left ventricular ejection fraction (LVEF), and right ventricular fractional shortening (RVFS). Sham *n* = 21–23; AoB: *n* = 20–23. *B*: pathophysiological sequelae of heart failure in the myocardial infarction (MI) mouse model. LV PSLAX B- and M-mode images with quantification of LV end-diastolic volume (EDV) as well as LVEF and RVFS. Control, *n* = 13–33; MI, *n* = 13–32. Female animals are highlighted by data points with purple border color. Data are represented means ± SE. Scale bar = 1 mm for all images. Unpaired *t* test or Mann−Whitney test are used where applicable.

To assess pulmonary and central venous congestion with ultrasound, we used two animal models of experimental HF. AoB rats presented with turbulent aortic flow, reduced LV ejection fraction (EF), and impaired RV fractional shortening (FS), indicative of advanced systolic global HF (Fig. 1*A*). MI mice, similarly, showed reduced LV EF and markedly increased LV volumes, whereas RV function was affected to a lesser extent compared with AoB rats (Fig. 1*B*).

**Figure 2.**
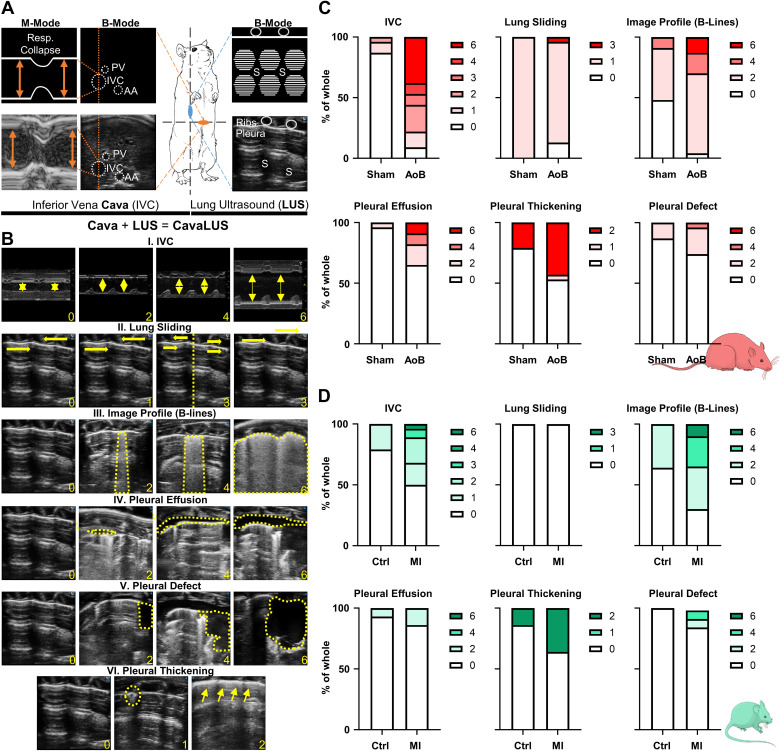
Ultrasonographic assessment of lung and inferior vena cava in experimental heart failure. *A*: lung ultrasound (LUS) and inferior vena cava (IVC) image acquisition. Portal vein (PV), IVC, and abdominal aorta (AA) were used as anatomical landmarks. An M-mode was placed across the IVC lumen (dotted line), and the maximum IVC diameter was measured (arrows). For LUS, the transducer was placed along the right anterior axillary line angled to 45° (blue mark). White hyperechoic pleural line in the upper image section was used as a visual landmark, accompanied by the subpleurally equidistantly stacked and spaced horizontal A-lines separated by rib shadows (S). *B*: sample images for IVC and LUS findings with severity scoring (points indicated in yellow). Related details can be found in Supplemental Table S1. *C* and *D*: frequency of IVC dilation and LUS-associated pathologies in sham (*n* = 23; all males) and AoB rats (*n* = 23; all males) (*C*) and in control (*n* = 13; 6 males, 7 females) and MI mice (*n* = 26; 7 males, 19 females) (*D*). MI, myocardial infarction.

To quantify congestion, we systematically imaged IVC diameters and LUS by assessing lung sliding, the presence of B-lines, pleural effusion, defects, and thickening (Fig. 2, *A* and *B*; Supplemental Table S1). Congestive imaging in AoB rats revealed a high incidence of IVC dilation, pulmonary edema-indicating B-lines, pleural effusion, thickening, and defects, whereas assessment of lung sliding was inconclusive with a lung pulse commonly present in both groups (Fig. 2*C*). In MI mice, the overall incidence of IVC dilation and LUS findings was lower compared with rats, but they also showed signs of IVC dilation, lung edema formation, and pleural defects compared with controls. Lung sliding was not properly assessable in mice (Fig. 2*D*).

**Figure 3.**
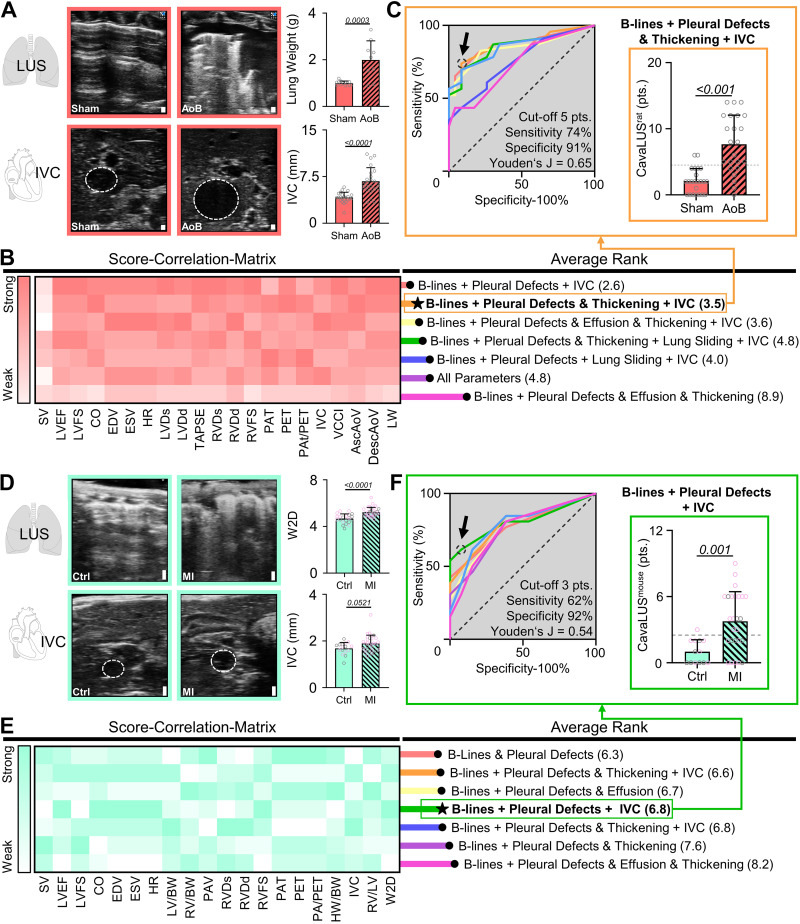
Development of species-specific CavaLUS scores. *A*: representative lung ultrasound (LUS) and inferior vena cava (IVC) B-mode images with gravimetric quantification of lung weight and maximum diameter of the IVC. Sham, *n* = 13–23; AoB, *n* = 9–23. *B*: score-correlation matrix evaluating the best performing CavaLUS^rat^. Red, strong correlation; white, weak correlation. *C*: receiver-operator characteristics, identifying the combination of B-lines, pleural defects, IVC size and pleural thickening (orange line) as most sensitive and specific for CavaLUS^rat^. Sham, *n* = 21–23; AoB, *n* = 20–23. *D*: LUS and IVC B-mode images with quantification of the lung wet weight-to-dry weight ratio (W2D) and maximum diameter of the IVC. Control, *n* = 16–21; MI, *n* = 31 or 32. *E*: score-correlation matrix evaluating the best performing CavaLUS^mouse^. Green, strong correlation; white, weak correlation. *F*: receiver-operator characteristics, identifying the combination of B-lines, pleural defects, and IVC diameter (green line) performed best for CavaLUS^mouse^. Control, *n* = 13–33; MI, *n* = 13–32. Female animals are highlighted by data points with purple border color. Data are represented as means ± SE. Scale bar = 1 mm for all images. Unpaired *t* test or Mann−Whitney test are used where applicable. SV, stroke volume; LVFS, left ventricular (LV) fractional shortening; CO, cardiac output; ESV, end-systolic volume; HR, heart rate; LVDs/d, systolic/diastolic LV diameter; TAPSE, tricuspid annular plane systolic excursion; RVDs/d, systolic/diastolic right ventricular (RV) diameter; VCCI, vena cava collapsibility index; Asc/DescAoV, ascending/descending aortic velocity; LW, lung weight; HW/BW, heart weight-to-body weight ratio; LV/BW, LV weight-to-BW ratio; RV/BW, RV weight-to-BW ratio; RV/LV, RV weight-to-LV weight ratio; PSLAX, parasternal long axis; PAT pulmonary acceleration time; PET pulmonary ejection time; pts., points.

Next, we confirmed the presence of pulmonary congestion in AoB rats by gravimetric assessment of lung weight (Fig. 3*A*). Central venous congestion was indicated by IVC dilation, evident as 60% diameter increase in AoB rats compared with sham controls. To formulate our novel congestion score, we investigated possible combinations of IVC assessment and LUS findings for their performance in assessing congestion. To this end, we developed a score-correlation matrix where all scored LUS signs together with IVC diameters (Fig. 2*B*, Supplemental Table S1) were correlated to key metrics of echocardiographically assessed HF or gravimetrically assessed pulmonary congestion (Fig. 3*B*). We assigned ranks to each combination based on their overall correlation performance (Fig. 3*B*, *right*), where lower ranks indicate better correlations. This approach enabled us to identify the combination of LUS signs and IVC diameters best qualified to assess congestion. The seven best performing combinations of IVC and LUS signs (Fig. 3*B*, *right*) were further investigated by receiver operator characteristics (ROC) analyses to determine the combination with the highest sensitivity and specificity at identifying HF animals with congestion (Fig. 3*C*). As a result, we found a combination of IVC diameter, LUS image profile (B-lines), pleural defects, and thickening most specific (91%) and sensitive (74%) to discriminate between sham animals and AoB rats with congestion (cutoff, 5 points; Youden’s *J* = 0.65). We refer to this combination hereinafter as CavaLUS^rat^ (Fig. 3*C* and Supplemental Table S2).

In MI mice, pulmonary edema formation was confirmed by increased lung wet weight-to-dry weight (W2D) ratios, whereas IVC diameters tended to be larger in MI mice, yet without statistical significance (*P* = 0.0521) (Fig. 3*D*). Following an equivalent approach as in rats, we used a score-correlation matrix, a rank system, and ROC analyses to identify the best-performing combination of IVC and LUS findings in mice (Fig. 3, *E* and *F*). The combination of IVC size, image profile (B-lines), and pleural defects reached high specificity (92%) and moderate sensitivity (62%) to discriminate controls from MI mice with congestion (cutoff, 3 points; Youden’s *J* = 0.54). Although the assessment of pleural thickening proved to add diagnostic value in rats, it did not in mice. We hereinafter refer to this as CavaLUS^mouse^ (Fig. 3*F*, Supplemental Table S2).

**Figure 4.**
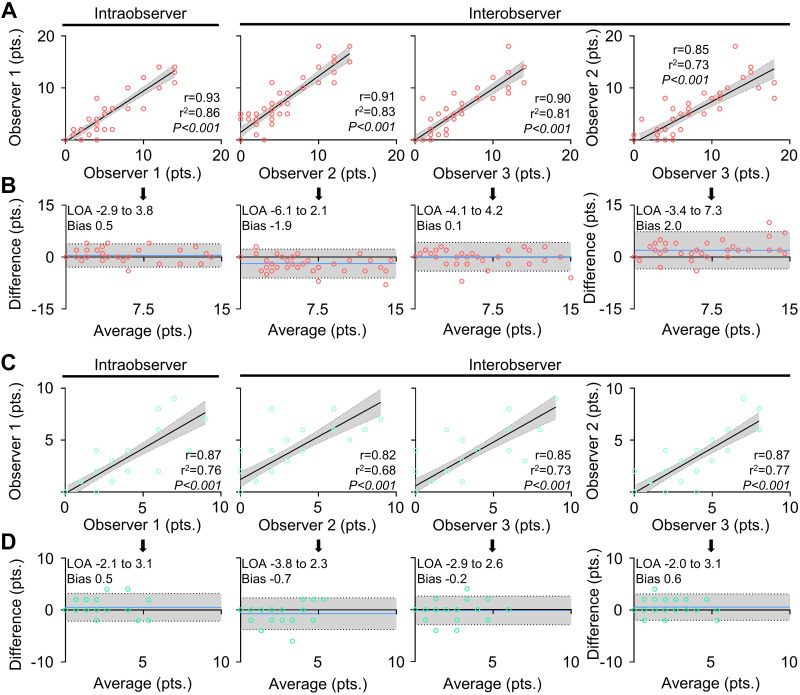
Intra- and interobserver analyses of CavaLUS^rat^ and CavaLUS^mouse^. *A* and *B*: intra- and interobserver analysis for CavaLUS^rat^ by Pearson’s correlation (*A*) with Blant−Altman plots for calculation of limits of agreement (95% LOA; gray area) and observer bias (blue line) (*n* = 46; all males) (*B*). *C* and *D*: intra- and interobserver analysis for CavaLUS^mouse^ by Pearson’s correlation (*C*) with Blant−Altman plots for calculation of limits of agreement (95% LOA; gray area) and observer bias (blue line) (*n* = 39; 13 males, 26 females) (*D*). pts., points.

Finally, we investigated observer agreements to investigate reproducibility of the image analysis by different sonographers (Fig. 4). Assessment of intra- and interobserver variability for CavaLUS^rat^ by Pearson’s correlation revealed strong observer agreement between three independent experienced observers with *r* ≥ 0.85 (Fig. 4*A*). In addition, Blant–Altmann plots indicated low bias for *observers 1* and *3* (0.1 and 0.5 points), whereas *observer 2* showed mild bias in both models (−1.9 and 2.0 points) (Fig. 4*B*). For CavaLUS^mouse^, similar results were seen with decent observer correlations showing *r* ≥ 0.82 and low bias between −0.7 and 0.6 points (Fig. 4, *C* and *D*).

## DISCUSSION

Here we introduce a novel, noninvasive tool to monitor systemic congestion in experimental HF in rodents. By integrating metrics of small animal LUS and IVC imaging, we identified the minimum combination of features needed to reliably identify pulmonary and central venous congestion (CavaLUS), thus abolishing the need to appraise all known LUS findings (10).

Clinical investigations using IVC and LUS assessment have proven superior to other laboratory tests and imaging modalities used for clinical decision-making in decompensated patients with HF (16). CavaLUS^mouse^ and CavaLUS^rat^ are largely identical (assessment of B-lines, pleural defects, and IVC size) with the only exception that pleural thickening contributed diagnostic power to the assessment of congestion in AoB rats. This discrepancy likely arises from the partial resistance of mice to PH development and as such, less central venous congestion in MI mice compared with AoB rats (17). Although congestion was less pronounced in MI mice, CavaLUS^mouse^ still yielded respectable diagnostic accuracy, marginally down from CavaLUS^rat^.

Moreover, intra- and interobserver variabilities were comparably low and agreement rates were high for both scores, overall performing comparably good as reported in a multitude of clinical studies using LUS and preclinical studies, executing conventional small animal echocardiography (18–21).

In conclusion, our findings demonstrate that combining LUS and IVC diameter assessment, CavaLUS, is superior to using LUS alone and enables the reliable, noninvasive, and repetitive assessment of the congestive sequelae in experimental HF.

## LIMITATIONS

Our study is limited by incomplete data sets as part of retrospective image analysis, affecting the overall power of the study; hence cautious data interpretation is warranted. Second, as the AoB cohort consisted only of male rats, our study is not suitable for assessment of sex-dependent differences of congestion in HF.

## DATA AVAILABILITY

All relevant data are included in the manuscript. Full data sets are available on request and will be made available by the authors, without undue reservation.

## SUPPLEMENTAL DATA

10.6084/m9.figshare.24598512 Supplemental Tables S1 and S2 and Supplemental Videos S1–S8: https://doi.org/10.6084/m9.figshare.24598512.

## GRANTS

J.G. was supported by the German Centre for Cardiovascular Research (DZHK), German Research Foundation (DFG) Grants SFB1470 A4 and GR 5261/1-1, the German Society for Cardiology (DGK), Corona-Stiftung Grant S199/10086/2022, and Dt. Stiftung für Herzforschung. W.M.K. was supported by DFG Grants SFB-TR84 A2 and C9, SFB-1449 B1, SFB 1470 A4, KU1218/9-1, KU1218/11-1, and KU1218/12-1; the German Ministry of Education and Research (BMBF) in the framework of SYMPATH (01ZX1906A) and PROVID (01KI20160A). M.K. was supported by the German Heart Foundation/German Heart Research Foundation (DSHF) Grant F23/20. C.K. was supported by DZHK and BMBF.

## DISCLOSURES

No conflicts of interest, financial or otherwise, are declared by the authors.

## AUTHOR CONTRIBUTIONS

N.H. and J.G. conceived and designed research; N.H., P.S., and J.G. performed experiments; N.H., J.H.K., and J.G. analyzed data; N.H. interpreted results of experiments; N.H. prepared figures; N.H. drafted manuscript; N.H., N.M., M.M.K., B.O., M.N., W.M.K., and J.G. edited and revised manuscript; N.H., J.H.K., C. Koçana, N.M., J.K., M.M.K., C. Knosalla, B.O., S.S., M.N., W.M.K., and J.G. approved final version of manuscript.
